# Predictors and Outcomes of Infection-Related Hospital Admissions of Heart Failure Patients

**DOI:** 10.1371/journal.pone.0072476

**Published:** 2013-08-23

**Authors:** Danny Alon, Gideon Y. Stein, Roman Korenfeld, Shmuel Fuchs

**Affiliations:** Internal Medicine B, Beilinson campus, Rabin Medical Center, Petach Tikva and Sackler School of Medicine, Tel Aviv University, Tel Aviv, Israel; National Institutes of Health, United States of America

## Abstract

**Background:**

Infections are one of the most common causes for hospitalization of patients with heart failure (HF). Yet, little is known regarding the prevalence and predictors of different types of acute infections as well as their impact on outcome among this growing population.

**Methods and Results:**

We identified all patients aged 50 or older with a major diagnosis of HF and at least one echocardiography examination who had been hospitalized over a 10-year period (January 2000 and December 2009). Infection-associated admissions were identified according to discharge diagnoses. Among 9,335 HF patients, 3530 (38%) were hospitalized at least once due to infections. The most frequent diagnoses were respiratory infection (52.6%) and sepsis/bacteremia (23.6%) followed by urinary (15.7%) and skin and soft tissue infections (7.8%). Hospitalizations due to infections compared to other indications were associated with increased 30-day mortality (13% vs. 8%, p<0.0001). These higher mortality rates were predominately related to respiratory infections (OR 1.28 [95% CI 1.09, 1.5]) and sepsis\bacteremia (OR 3.13 [95% CI 2.6, 3.7]). Important predictors for these serious infections included female gender, chronic obstructive pulmonary disease, past myocardial infarction and echocardiography-defined significant right (RV) but not left ventricular dysfunction.

**Conclusions:**

Major infection-related hospitalizations are frequent among patients with HF and are associated with increased mortality rates. Elderly female patients with multiple comorbidities and those with severe RV dysfunction are at higher risk for these infections.

## Introduction

Heart failure (HF) is a leading cause of hospitalization among the elderly over 65 in western countries. [1], [2] Such hospitalizations are associated with substantial mortality rates, due to both HF exacerbation and non-HF conditions. [3] Infections, predominantly respiratory infections are among the main co-morbidities diagnosed in hospitalized HF patients, ranging from 2.5% for pneumonia to 4.5% for chronic obstructive pulmonary disease (COPD). [2], [4], [5] In the Organized Program to Initiate Lifesaving Treatment in Hospitalized Patients With Heart Failure (OPTIMIZE-HF) the rate of pneumonia/respiratory processes was identified as a precipitating factor in 15.3% of the patients and these were associated with 1.6 fold increase in hospital mortality. [6] A recent analysis of Canadian national hospital admissions data revealed that pneumonia was the fourth most common non-cardiovascular co-morbidity among patients hospitalized for HF, affecting 2.5% of the patients. Furthermore, HF was listed as a co-morbid condition in 5.8% of patients who were hospitalized for the primary diagnosis of pneumonia. [4] Despite these observations, there is a paucity of data regarding the frequency of different infection types, risk factors associated with the development of these infections and their impact on short-term mortality, in HF patients. Accordingly, we sought to determine the prevalence, clinical impact and predictors for major infections related to hospitalizations among patients with HF and their associations with short-term mortality.

## Materials and Methods

### Identification of Patients

All consecutive patients, with a major diagnosis of chronic HF or a diagnosis of HF on discharge (the ICD-9 codes used for the identification of HF diagnoses are listed in Table S1), admitted to the Rabin Medical Center, Petah Tikva, Israel, between January 1^st^ 2000 and December 31^st^ 2009. Patients were included if they were ≥50 years old at the time of their first admission and had at least one echocardiography examination available. Patients who underwent valve replacement surgery during the study period were excluded.

### Data Extraction

Two separate hospital computerized electronic records were used in this study: 1) Rabin Medical Center’s electronic medical records system (EMR) that includes patient demographics and hospitalization-related parameters, and 2) An echocardiography database that includes examination dates and detailed echocardiographic measurements. All data was obtained from the last available examination.

Clinical data collected included age, gender, date of death and ICD-9 codes of chronic diagnosis including: diabetes, hypertension, hyperlipidemia, chronic renal failure, malignancy, atrial fibrillation, obesity, history of myocardial infarction (MI), ischemic heart disease, history of cerebrovascular accident (CVA), COPD and anemia. Data on hospitalizations included admission and discharge dates, and ICD-9 codes of the main discharge diagnosis. Types of infections included in the study were: pneumonia, COPD exacerbation, urinary tract infection (UTI), skin and soft tissue infections, sepsis and bacteremia. Data on infections were based on the main discharge diagnosis.

### Definition of Covariates

Left ventricular (LV) function was classified to: normal (ejection fraction (EF)> = 55), mild dysfunction (EF 45–54), moderate dysfunction (EF 30–44) and severe dysfunction (EF <30). [7] Heart failure with reduced ejection fraction (HFREF) was defined as HF with ejection fraction (EF)≤45% (mild-to-moderate LV dysfunction or worse). Heart failure with preserved ejection fraction (HFPEF) was defined as HF with EF>45% (normal or mildly reduced LV function). Severe diastolic dysfunction was defined as restrictive LV filling and categorized, according to the presence or lack of its reversibility at peak Valsalva maneuver, as grade 3 (reversible restrictive pattern) or grade 4 (non-reversible restrictive pattern) dysfunctions. [8] Heart failure associated with significant valvular disease was defined as HF in the presence of 1) aortic stenosis with mean gradient of >40 mmHg; 2) aortic, mitral or tricuspid regurgitation of moderate severity or worse. Valvular disease of milder severity was considered insignificant. Significant left ventricular hypertrophy (LVH) was defined as moderate left ventricular posterior wall (LVPW), or interventricular septum thickness of 14 mm or more. [7].

Pulmonary hypertension was defined as peak tricuspid regurgitant velocity on Doppler echocardiography of 3.0 m/sec that corresponds (assuming right arterial pressure of 5 mmHg) to a peak systolic pulmonary arterial pressure of 40 mmHg. Annualized admission rate was determined as the ratio between the number of admissions and years of follow-up.

In order to explore the true burden of re-hospitalization, we excluded from the calculation of readmissions at 30-days and 6-months and from the annualized admission rates at 1-year, all patients who either died prior or were not followed-up for the pre-specified period.

The study was approved by Rabin Medical Center’s institutional ethics committee. As this was a retrospective study, and the investigators were blinded to the personal details of the patients. The institutional ethics committee waived the need for an informed consent.

### Mortality Data

Survival status was determined from the Rabin Medical Center registry that is updated monthly by the Israeli Ministry of Internal Affairs registry. Overall mortality was defined as death from all causes commencing from the first hospitalization until the end of the study period (December 31^st^, 2009). Thirty-day and one-year morality rates were calculated as mortality at the defined follow-up period commencing from the first admission.

### Statistical Analysis

Statistical analysis was performed with SPSS software for Windows (SPSS Inc., Chicago, IL, USA); continuous variables are expressed as a mean ±1 standard deviation (SD) and as a median [interquartile range] when indicated. Categorical variables are presented as percentages. Comparisons between the two groups were performed by Student’s *t*-test for continuous variables and the chi-square test for comparison of categorical values. All tests of significance were performed using two-tails with p value <0.05 considered significant. Multivariate analysis for predictors of severe infection was performed using multinominal logistic regression.

## Results

Data was obtained for 14,934 patients of whom 5,599 were excluded: 3,965 had no documented echocardiography study, 1,097 patients underwent valve replacement and 537 patients were under 50 years old. The final study cohort comprised of 9,335 patients with a near normal age distribution. During a followed-up period of 2.8±2.6 years, 3,530 patients (38% of the study cohort) experienced at least one infection-related hospital admission.

### Baseline Patient Characteristics

Mean age of the cohort was 76±10 years (median 77, inter-quartile range 69–83). Ninety two percent of the patients had cardiovascular diseases and associated risk factors and 72% had non-cardiovascular co-morbidities. The main causes for infection-related hospitalizations were: respiratory (including pneumonia and COPD exacerbations –52.6%), sepsis and bacteremias (23.6%), UTI (15.7%) and skin and soft tissue (7.8%). Patients who were admitted due to infections were significantly older and had more cardiovascular and non-cardiovascular co-morbidities (Table 1).

**Table 1:** Patient characteristics.

Parameter	No Infection (N = 5805)	Any Infection (N = 3530)	p
Age (avg±stdev)	75±10	77±10	<0.0001
Male (%)	57	55	NS
Diabetes (%)	41	44	0.004
CRF (%)	30	45	<0.0001
Malignancy (%)	14	16	NS
AFIB (%)	38	47	<0.0001
Obesity (%)	11	13	0.007
IHD (%)	67	69	NS
Past CVA (%)	13	20	<0.0001
COPD (%)	13	31	<0.0001

### Echocardiographic Characteristics

Echocardiographic measures differed significantly between HF patients with infection-related versus non-infectious related admissions. Patients with infection-related admissions had more frequently right ventricular dysfunction (22% vs. 18%, p = 0.0002). This significant increased rate was noted for all types of infections with the exception of urinary tract infections. Patients who experienced sepsis and bacteremia had, in addition to right ventricular dysfunction, higher rates of systolic (45% vs. 40%, p = 0.007) and diastolic HF (30% vs. 18%, p = 0.0004) as well as higher rates of significant valvular abnormalities (37% vs. 31%, p = 0.002).

### Recurrent Admissions

Infection-related admissions accounted for 24% of all admissions and 52% of all non-HF related admissions. Patients admitted for infection related causes had an increased rate of readmissions at 6 months, compared to non-infection related admissions (65% vs. 33%, p<0.0001). Interestingly, those admitted due to infections had higher rates of annualized admission due to HF exacerbation (0.97±1.14 vs. 0.61±0.71, p<0.0001).

### Mortality

Thirty-day mortality rate was 10% for the entire cohort. Patients admitted for infectious causes had significantly higher 30-day (13% vs. 8%, p<0.0001) and one-year (69% vs. 37%, p<0.0001) mortality rates.

**Figure 1:**
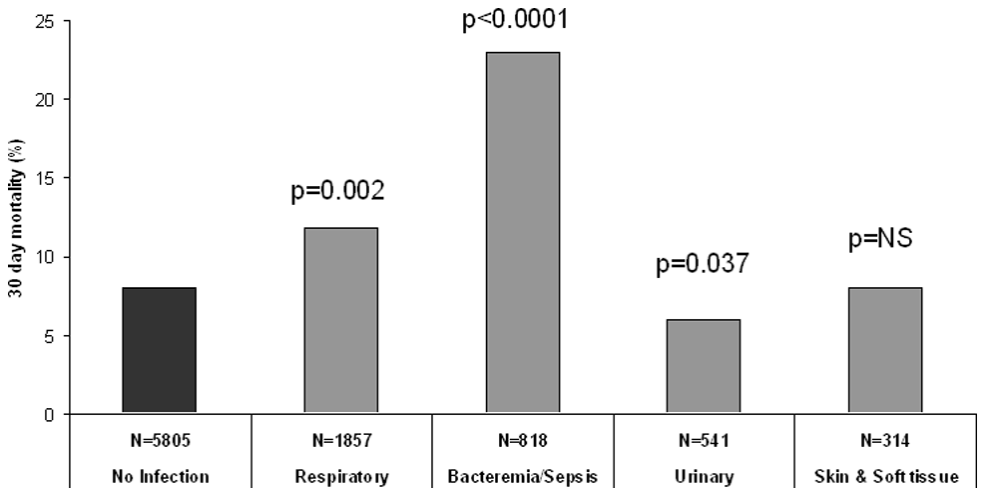
30-day mortality rates per infection type. Thirty-day mortality was compared between each of the HF patient cohorts admitted with distinct infections and HF patients admitted for non-infectious causes. HF patients admitted with respiratory infections, bacteremia or sepsis had significantly higher mortality rates, while HF patients admitted with skin and urinary tract infections had similar and lower 30-day mortality rates, respectively.

Analysis by infection type showed that patients admitted for a non-infectious cause, compared to those admitted for sepsis/bacteremia and respiratory infections had significantly higher 30-day mortality rates (Figure 1).

### Predictors for Severe Infection Related Admissions

Predictors for infections associated with increased short-term mortality (respiratory and sepsis/bacteremia) are listed in Table 2. COPD was found to be the strongest clinical predictor and significant RV dysfunction was found to be the single echocardiographic measure associated with high-risk infections (Table 2).

**Table 2:** Predictors for respiratory infection sepsis or bacteremia.

Parameter	OR (CI 95%)	p
Age	1.02 (1.01, 1.03)	0.004
Male gender	0.77 (0.61, 0.96)	0.022
Chronic renal failure	1.49 (1.18, 1.87)	0.001
Malignancy	1.58 (1.19, 2.1)	0.001
Atrial fibrillation	1.67 (1.32, 2.12)	<0.0001
Past Myocardial Infarction	1.95 (1.5, 2.54)	<0.0001
Past CVA	1.52 (1.14, 2.02)	0.004
COPD	4.87 (3.83, 6.2)	<0.0001
Anemia	1.64 (1.31, 2.06)	<0.0001
Significant RV dysfunction	1.41 (1.05, 1.89)	0.022

### The Impact of HF on Infection Related Admissions

To assess the impact of HF on infection-related admissions we analyzed an additional cohort of 14,774 consecutive patients, aged ≥50 years old, who were hospitalized in Rabin Medical Center, Israel, between July 2011 and December 2012 (23,413 admissions). Patients with HF comprised 16.5% (n = 2442) of the cohort and compared to patients without HF, were older (79±10 vs. 73±12, p<0.0001) yet there was a similar gender distribution (52% vs. 50% males, p = NS). Patients with HF, compared to those without HF, had a higher incidence of infection-related admissions (32% vs. 21%, p<0.0001). Among patients who were admitted due to infection, the relative frequency of each infection type differed between patients with and without HF. Respiratory infections were more common among HF patients (43% vs. 31%, p<0.0001), urinary tract infections were more common among non-HF patients (38% vs. 49%, p<0.0001) and the frequency of bacteremia/sepsis was similar (34% vs. 31%, p = NS).

Thirty-day mortality rates overall were significantly higher among patients with HF compared to those without HF (13% vs. 8%, p<0.0001). These differences were mostly due to a 25% increase in the mortality rate among HF patients who were admitted with bacteremia/sepsis (51% vs. 41%, p = 0.015). There were no statistically significant differences in thirty-day mortality among patients with and without HF who were admitted for pulmonary (16% vs. 12%, p = NS) and urinary tract infections (15% vs. 11%, p = NS).

## Discussion

The current study assessed the clinical and echocardiographic measures, predictors and outcome regarding infection-related hospital admission of patients with HF. The main study findings indicate: 1) Infections represent a substantial burden on HF related hospitalizations, 2) respiratory infections and bacteremia/sepsis -related hospitalization compared to other indications are associated with increased mortality rates 3) female patients and those with COPD as well as other co-morbidities are at increased risk for severe infections and 4) serious infections among HF patients are related to the severity of RV rather than LV failure.

In the current study, 38% of the patients experienced at least one hospital admission due to an infection, which accounted for 24% of all admissions. Previous studies focused mainly on assessing the rates of pneumonia as a precipitating factor for HF exacerbations, with reported rates of 6.6%–35.4%. [4], [9] Although we observed a similar rate of respiratory infections (15%), these infections accounted for only half of all infection-related admissions. Accordingly, our observation suggests that the true burden of infection-related admission is substantially higher than previously reported.

Short-term mortality rates of hospitalized HF patients vary, with reported rates ranging between 2–20%. [1] We observed an overall similar 30-day mortality rate of 10%. Nevertheless, admissions due to infections were associated with a 60% increase in short-term mortality, predominately related to respiratory and systemic infections. This observation emphasizes the significance of infections in HF patients as both a well-established precipitator for acute decompensated HF and as a direct contributor for mortality.

The worsened outcome associated with respiratory and systemic infections compared to other indications for hospital admission, may reflect the complex and vicious interaction between HF and infection. Respiratory infections, for example, have been demonstrated as a major trigger for cardiac complications, affecting more than a quarter of those hospitalized with community acquired pneumonia. [10] The acute systemic inflammation associated with the response to infection can directly depress myocardial function as well as alter the delicate balance of oxygen demand and supply. [11]–[13] Such interplay is well known in sepsis and bacteremia and involves mechanistic pathways, which are also common in acute decompensated HF. In addition, an increase in pulmonary pressures and the direct myocardial depressant effect of pneumonia related pathogens, such as streptococcal pneumonia, Haemophilus influenza, Mycomplasma pneumoniae, Chlamydophila pneumonia and Legionella pneumophila, may all carry a continuous deleterious impact. [14]–[17] It is thus conceivable that infection-related unwarranted cardiac and systemic effects differ from other, “time-limited” triggers of acute HF decompensation, such as arrhythmia, bleeding or acute withdrawal of medications. These distinctions may explain, at least in part, the worsened outcomes of HF patients hospitalized due to infections.

The increased mortality among patients hospitalized for infections was due to adverse outcomes associated with respiratory and systemic infections. These were also the most common infections in our study, accounting for 75% of all infections. Interestingly, the single identified echocardiographic predictor for these major infections was RV dysfunction. Previous studies suggest that HF is a risk factor for community-acquired pneumonia among patients with COPD. [18] The importance of significant RV dysfunction among hospitalized patients with HF was also recently reported in both young and elderly patients. [3] Possibly the predictive value of significant RV dysfunction in our patient population mirrors the increased severity of combined cardiac and pulmonary disease. Whether the severity of RV failure is a marker or a contributing cause of respiratory and systemic infection is an important issue, which the current study was not designed to address.

In the current study, using an additional cohort we found that patients with HF, compared to those without HF had an almost 50% increase in infection-related admissions and a 62% increase in 30-day mortality. It is conceivable that these increased mortality rates are related, at least in part, to advanced age and to pre-existing HF, which in the setting of systemic infection adversely affect hemodynamics and contribute to the increased rates of cardiac related complications. [10] Furthermore, we found that the relative frequency of various infection types differed between patients with HF compared to those without HF. Patients with HF had relatively higher rates of pneumonia and lower rates of urinary tract infections. The study was not designed to identify factors associated with the distinct distribution of infections among patients with and without HF, thus additional large cohort studies are warranted.

The main limitation of our study is its retrospective design with reliance on ICD-9 and discharge diagnoses rather than on direct clinical data. We were therefore unable to determine, for example, whether patients who experienced infection-related admission were given appropriate vaccinations or had any other infection-related predisposing factors such as nursing home residency. Nevertheless, a major strength of the study is its accurate characterization of HF status using echocardiographic variables available for all participating patients.

In conclusion, the burden of infection-related hospital admission among patients with HF is associated to several types of infections and is significant as compared to non-infectious causes, with increased short-term mortality. Clinical and echocardiographic measures can be used for identifying HF patients at increased risk for major infections, thus providing a tool for risk stratification, prevention and guidance for acute hospital care.

## Supporting Information

Table S1 **ICD-9 codes associated with heart failure.**
(DOC)

## References

[1] Fonarow GC (2008) Epidemiology and risk stratification in acute heart failure. Am Heart J 155: 200–207. 18215587 10.1016/j.ahj.2006.10.043

[2] Sarmento PM, Fonseca C, Marques F, Ceia F, Aleixo A (2006) Acutely decompensated heart failure: characteristics of hospitalized patients and opportunities to improve their care. Rev Port Cardiol 25: 13–27. 16623353

[3] Stein GY, Kremer A, Shochat T, Bental T, Korenfeld R, et al (2012) The diversity of heart failure in a hospitalized population: the role of age. J Card Fail 18: 645–653. 22858081 10.1016/j.cardfail.2012.05.007

[4] Dai S, Walsh P, Wielgosz A, Gurevich Y, Bancej C, et al (2012) Comorbidities and mortality associated with hospitalized heart failure in Canada. Can J Cardiol 28: 74–79. 21885240 10.1016/j.cjca.2011.05.002

[5] Castro P, Vukasovic JL, Garces E, Sepulveda L, Ferrada M, et al (2004) [Cardiac failure in Chilean hospitals: results of the National Registry of Heart Failure, ICARO]. Rev Med Chil 132: 655–662. 15332366 10.4067/s0034-98872004000600001

[6] Fonarow GC, Abraham WT, Albert NM, Stough WG, Gheorghiade M, et al (2008) Factors identified as precipitating hospital admissions for heart failure and clinical outcomes: findings from OPTIMIZE-HF. Arch Intern Med 168: 847–854. 18443260 10.1001/archinte.168.8.847

[7] Lang RM, Bierig M, Devereux RB, Flachskampf FA, Foster E, et al (2006) Recommendations for chamber quantification. Eur J Echocardiogr 7: 79–108. 16458610 10.1016/j.euje.2005.12.014

[8] Daneshvar D, Wei J, Tolstrup K, Thomson LE, Shufelt C, et al (2010) Diastolic dysfunction: improved understanding using emerging imaging techniques. Am Heart J 160: 394–404. 20826245 10.1016/j.ahj.2010.06.040

[9] Shafazand M, Patel H, Ekman I, Swedberg K, Schaufelberger M (2012) Patients with worsening chronic heart failure who present to a hospital emergency department require hospital care. BMC Res Notes 5: 132. 22401538 10.1186/1756-0500-5-132 PMC3315737

[10] Corrales-Medina VF, Musher DM, Wells GA, Chirinos JA, Chen L, et al. Cardiac complications in patients with community-acquired pneumonia: incidence, timing, risk factors, and association with short-term mortality. Circulation 125: 773–781. 22219349 10.1161/CIRCULATIONAHA.111.040766

[11] Colombo PC, Ganda A, Lin J, Onat D, Harxhi A, et al (2012) Inflammatory activation: cardiac, renal, and cardio-renal interactions in patients with the cardiorenal syndrome. Heart Fail Rev 17: 177–190. 21688186 10.1007/s10741-011-9261-3 PMC3876739

[12] Maeder M, Fehr T, Rickli H, Ammann P (2006) Sepsis-associated myocardial dysfunction: diagnostic and prognostic impact of cardiac troponins and natriuretic peptides. Chest 129: 1349–1366. 16685029 10.1378/chest.129.5.1349

[13] Egorova EN, Kalinkin MN, Mazur ES (2011) [Endotoxinemia and systemic inflammation in pathogenesis of chronic heart failure]. Patol Fiziol Eksp Ter: 42–46. 22359933

[14] Wang G, Burczynski F, Hasinoff B, Zhong G (2002) Infection of myocytes with chlamydiae. Microbiology 148: 3955–3959. 12480899 10.1099/00221287-148-12-3955

[15] Armengol S, Domingo C, Mesalles E (1992) Myocarditis: a rare complication during Legionella infection. Int J Cardiol 37: 418–420. 1468829 10.1016/0167-5273(92)90276-9

[16] Mamas MA, Fraser D, Neyses L (2008) Cardiovascular manifestations associated with influenza virus infection. Int J Cardiol 130: 304–309. 18625525 10.1016/j.ijcard.2008.04.044

[17] Paz A, Potasman I (2002) Mycoplasma-associated carditis. Case reports and review. Cardiology 97: 83–88. 11978954 10.1159/000057677

[18] Mullerova H, Chigbo C, Hagan GW, Woodhead MA, Miravitlles M, et al (2012) The natural history of community-acquired pneumonia in COPD patients: a population database analysis. Respir Med 106: 1124–1133. 22621820 10.1016/j.rmed.2012.04.008

