# Supplementary material for: Predictors and Outcomes of Infection-Related Hospital Admissions of Heart Failure Patients
Source: PLoS One. 2013 Aug 23;8(8):e72476. doi: 10.1371/journal.pone.0072476 (PMC3751916; doi:10.1371/journal.pone.0072476)
Supplement: Table S1 — ICD-9 codes associated with heart failure. (DOC) [file pone.0072476.s001.doc]

## Supplemental Data

### Table S1: ICD-9 codes associated with heart failure

| **ICD-9 code** | **Diagnosis** |
| --- | --- |
| 428 | HEART FAILURE |
| 514 | PULMONARY CONGESTION AND HYPOSTASIS |
| 428.0 | CONGESTIVE HEART FAILURE |
| 428.1 | LEFT HEART FAILURE |
| 428.2 | SYSTOLIC HEART FAILURE |
| 428.9 | HEART FAILURE, UNSPECIFIED |
| 518.4 | ACUTE EDEMA OF LUNG, UNSPECIFIED |
| 428.20 | SYSTOLIC HEART FAILURE, UNSPECIFIED |
| 428.21 | ACUTE SYSTOLIC HEART FAILURE |
| 428.30 | DIASTOLIC HEART FAILURE, UNSPECIFIED |
| 428.31 | ACUTE DIASTOLIC HEART FAILURE |
| 428.32 | CHRONIC DIASTOLIC HEART FAILURE |
| 428.33 | ACUTE ON CHRONIC DIASTOLIC HEART FAILURE |
| 428.40 | COMBINED SYSTOLIC AND DIASTOLIC HEART FAILURE, UNSPECIFIED |
